# High Precision Prediction of Functional Sites in Protein Structures

**DOI:** 10.1371/journal.pone.0091240

**Published:** 2014-03-14

**Authors:** Ljubomir Buturovic, Mike Wong, Grace W. Tang, Russ B. Altman, Dragutin Petkovic

**Affiliations:** 1 Department of Computer Science, San Francisco State University, San Francisco, California, United States of America; 2 Center for Computing for Life Sciences, San Francisco State University, San Francisco, California, United States of America; 3 Department of Bioengineering, Stanford University, Stanford, California, United States of America; Miami University, United States of America

## Abstract

We address the problem of assigning biological function to solved protein structures. Computational tools play a critical role in identifying potential active sites and informing screening decisions for further lab analysis. A critical parameter in the practical application of computational methods is the precision, or positive predictive value. Precision measures the level of confidence the user should have in a particular computed functional assignment. Low precision annotations lead to futile laboratory investigations and waste scarce research resources. In this paper we describe an advanced version of the protein function annotation system FEATURE, which achieved 99% precision and average recall of 95% across 20 representative functional sites. The system uses a Support Vector Machine classifier operating on the microenvironment of physicochemical features around an amino acid. We also compared performance of our method with state-of-the-art sequence-level annotator Pfam in terms of precision, recall and localization. To our knowledge, no other functional site annotator has been rigorously evaluated against these key criteria. The software and predictive models are incorporated into the WebFEATURE service at http://feature.stanford.edu/wf4.0-beta.

## Introduction

In the past decade, the amount of three-dimensional structural information for biological macromolecules has increased greatly, partly through technological advances as well as through the structural genomics initiatives that have prioritized the systematic determination of protein and nucleic acid structures [1] using X-ray crystallography, Nuclear Magnetic Resonance, electron microscopy, and other methods. As a result of this great acceleration of new information about 3D structure of proteins, there is a shift in the amount of background biological information available for many of the newly solved structures. In particular, there are many solved structures with no reported biological function, and so computational methods are critical to identify active sites and understand their molecular function. Methods based on sequence analysis are very powerful in this regard, as they can recognize domains and 1D motifs associated with function. Sometimes, however, only an analysis of the 3D structure allows the recognition of spatial interactions that are not apparent in the sequence analysis. Several methods have been developed to seek functional sites using 3D information including FFFs [2], TESS [3], GASPS [4], MarkUs [5] and FEATURE [6], [7].

An important protein function annotation strategy includes computational functional site prediction followed by experimental confirmation of the most promising results. In this context, the precision, or positive predictive value of the predictor is of paramount importance. This parameter quantifies the proportion of positive predictions which are indeed functional. Low precision models waste resources spent on laborious pursuit of functional activity that is not present. We postulate that an annotator which delivers at least 99% precision should have considerable utility in many realistic applications, such as identification of therapeutic targets. At this level of precision, ninety nine out of a hundred predicted functional sites would have been confirmed in the lab, and the challenge becomes maximizing recall (proportion of true functional sites found by the algorithm) among candidate computational models. Thus, the best method in the scenario we are considering maximizes recall at 99% precision. To our knowledge, none of the previously proposed sequence-based or structure-based methods had been developed for or rigorously evaluated against these specific goals, and thus this presented a key motivation for the present work.

The basis for our approach was FEATURE, a function annotation method that uses 3D protein structure information. FEATURE regards functional sites as protein microenvironments represented by vectors of physicochemical properties (features). For developing machine learning predictors, these vectors are aggregated to build Naïve Bayes classification models for recognizing the location of binding and active sites by using examples of these sites of interest as a positive training set (e.g. calcium binding sites [8], [9], or thioredoxin active sites [10]) and using suitable non-sites as the negative training set. In this paper we utilized the FEATURE vectors and Support Vector Machine learning algorithm to construct a functional annotator which meets the stated precision and recall goals. The classifier choice was based on comprehensive evidence of SVM performance [11], [12], availability of industrial-strength software library [13] and the authors’ own experience [14], [15]. We also compared the new FEATURE with Pfam [16], a sequence-based annotator commonly used for functional annotation.

## Methods

### Materials

We built a 3D annotator FEATURE, which assigns functional sites defined in PROSITE [17] to novel protein structures. We used Protein Data Bank (PDB) [18] as the source of protein structures and PROSITE as the source of protein functional site definitions for supervised training of FEATURE machine learning models. PROSITE patterns are manually curated and are created according to previous observations from literature or from a sequence alignment of the protein sequences possessing the observed function. The patterns are derived from the alignment by taking the shortest common subsequence that matches known proteins with high specificity. Each pattern may result in multiple FEATURE predictive models, one for each functional atom in a conserved residue. Crucially, PROSITE entries identify *true positive* and *false positive* examples. It is this information which enabled us to conduct accurate learning and evaluation of the FEATURE functional predictive models.

Each FEATURE model requires positive and negative examples for training. We considered a structure to be a *positive* example if PROSITE indicated that it contained the functional site being modeled. Structures were considered *negative* examples if they were not positive. The positive and negative examples were chosen as follows:

• Positive examples
  1. Identify true positive PROSITE examples and extract their structure data from the PDB. To avoid redundancy, cluster homologs sharing 100% sequence similarity and select a single representative structure with the highest X-ray crystallography resolution from each cluster for further processing.
  2. For each of the PDB structures, map the PROSITE pattern to the amino acid sequence of the protein and find the residue number and residue name of the conserved residues in the PDB protein sequence.
  3. Extract coordinates of functional atoms for all residues identified in Step 2b. The different conserved residues represent positive examples for the given predictive model; the extracted coordinates of the functional atoms are used to calculate feature vectors for training the FEATURE classifiers.
• Negative examples
  1. From a snapshot of all PDB structures available at the time of PROSITE 20.80 release, remove structures that are associated with the given functional class, as identified by PROSITE (i.e. we removed positive or potentially positive examples). We did not take negative examples from proteins containing positive sites, in order to avoid possible contamination of the negative set with sites that are close to positive sites and therefore contain residual signal.
  2. For each functional atom in a PROSITE pattern, find 50,000 atom coordinates by randomly choosing atoms within remaining PDB structures with the same residue name and atom name, sampling without replacement.

All positive and negative coordinates were converted to FEATURE vectors to generate the positive and negative samples for training the models. Specifically, we used *Featurize*, a function available in the public release of the FEATURE package (https://simtk.org/home/feature). *Featurize* extracts physicochemical properties from the three-dimensional structure of the spatial neighborhood surrounding the position associated with the function of interest. It represents functional sites as protein microenvironments that contain six spherical shells of 1.25 Ångstroms in thickness, oriented around a central point of interest. *Featurize* accumulates statistics about the abundance of atoms, residues, secondary structures, charge, polarity, hydrophobicity and other biophysical and biochemical properties (totaling 80 properties in each shell) in order to describe a microenvironment in a vector of 6 shells×80 properties = 480 features. The characteristic properties are represented as numeric vectors and are listed in Table 1.

**Table 1:** List of physicochemical properties used to characterize a functional site.

Property Type	Property Name
**AtomName**	C, N, O, S, ANY, OTHER
**ChemicalGroup**	Hydroxyl, Amide, Amine, Carbonyl, RingSystem, Peptide
**AtomProperties**	VDWVolume, Charge, Hydrophobicity, Mobility, Solvent Accessibility
**ResidueName**	ALA, ARG, ASN, ASP, CYS, GLN, GLU, GLY, HIS, ILE, LEU, THR, LYS, MET, PHE, PRO, SER, TRP, TYR, VAL, HOH, OTHER
**ResidueProperties**	Hydrophobic, Charged, Polar, NonPolar, Basic, Acidic
**SecondaryStructure**	3Helix, 4Helix, 5Helix, Bridge, Strand, Turn, Bend, Coil, Het, Unknown

We chose 20 biologically distinct protein models based on adequate number of positive examples, biological relevance as judged by the authors and available resources for analyses. The choice was made prior to any downstream processing and never changed. The training samples for each protein model were converted to vectors of physicochemical properties using *Featurize*. The details of the protein models are given in Table 2.

**Table 2:** Functional families used to evaluate performance of FEATURE.

PROSITE	Index	Amino-acid	Atom
**ADH_SHORT**	5	TYR	eta oxygen (OH)
**ALPHA_CA_1**	11	HIS	epsilon nitrogen #2 (NE2)
**ASP_PROTEASE**	4	ASP	delta oxygen #2 (OD2)
**ATPASE_ALPHA_BETA**	8	SER	gamma oxygen (OG)
**CARBOXYLESTERASE_B_1**	3	CYS	gamma sulfur (SG)
**CYTOCHROME_P450**	8	CYS	gamma sulfur (SG)
**EF_HAND**	1	ASP	delta oxygen #1 (OD1)
**EGF_1**	10	CYS	gamma sulfur (SG)
**IG_MHC**	3	CYS	gamma sulfur (SG)
**INSULIN**	2	CYS	gamma sulfur (SG)
**LACTALBUMIN_LYSOZYME**	3	CYS	gamma sulfur (SG)
**LECTIN_LEGUME_BETA**	6	ASP	delta oxygen #1 (OD1)
**PA2_HIS**	2	HIS	gamma sulfur (SG)
**PROTEIN_KINASE_ST**	5	ASP	delta oxygen #2 (OD2)
**PROTEIN_KINASE_TYR**	5	ASP	delta oxygen #2 (OD2)
**RNASE_PANCREATIC**	2	LYS	zeta nitrogen (NZ)
**SOD_CU_ZN_1**	3	HIS	epsilon nitrogen #2 (NE2)
**TRYPSIN_HIS**	5	HIS	epsilon nitrogen #2 (NE2)
**TRYPSIN_SER**	6	SER	gamma oxygen (OG)
**ZINC_PROTEASE**	5	GLU	epsilon oxygen #1 (OE1)

Column PROSITE lists functional families used to evaluate performance of FEATURE. Column Index is index of the conserved position within the corresponding PROSITE regular expression. Column Amino-acid is code of the amino-acid at that position. Column Atom is the residue atom at which the FEATURE microenvironment is centered.

We note that PROSITE also provides *false negative* designation for certain PDB proteins, which could in principle be used as positive examples. In practice, this is challenging because these proteins are known to have the function, yet do not conform to the PROSITE pattern and thus the exact atomic coordinates of the functional site are not available through PROSITE/PDB. This in turn prevents FEATURE modeling since it requires exact location of the functional site, and consequently we did not use false negatives in any analyses.

### Classifiers

The FEATURE concept consists of multivariate representation of functional sites using the physicochemical microenvironment properties as feature vectors, followed by a classifier which assigns function (or lack thereof) to the resulting vector of properties. The original FEATURE system [6], [7] used the Naïve Bayes classifier, whereas the focus of this work is the Support Vector Machine classifier. To distinguish the two, we refer to them as FEATURE-SVM and FEATURE-NB.

#### Support vector machine

The Support Vector Machine classifier refers to several variations of a two-class linear classifier described as having the *maximum margin* property. Intuitively, the property means that the linear classification hyperplane is as distant as possible from training data points in both classes.

In standard formulation, SVM is a linear two-class classifier over a feature vector *x*

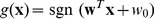
(1)where the coefficients 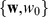 are chosen to yield the maximum margin by solving the following constrained optimization problem:



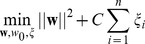
(2)

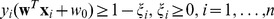



Here, 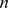 is total number of positive and negative examples in the training set, 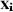 are the feature vectors, and 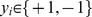 are their class labels. 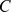 is a user-defined positive constant and 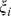 measures the degree of misclassification of example 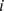. Large values of 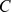 improve training data accuracy paid for by decreased generalization ability of the classifier.

This problem is equivalent to the standard linear regression problem [19], [20]

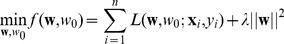
(3)where 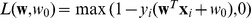 is the *hinge loss* term, the second term is the regularization term, and 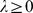 a user-given constant. The loss term measures accuracy of the classifier on the training data; the regularization term controls the generalization ability of the classifier. The constant 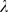 controls the trade-off between the two goals. The hinge loss distinguishes Support Vector Machine from other linear regression algorithms.

In this paper we used formulation (2).

In particular for this application, it is critically important to generate class-conditional posterior probabilities because they drive the decision of whether to invest scarce resources into experimental confirmation of putative functional sites. The Naïve Bayes algorithm used in FEATURE-NB natively produces the posterior probabilities. However, in original formulation, the SVM algorithm does not produce the probabilities, but scores on an arbitrary, non-intuitive scale. To overcome this issue, we used the probabilistic extension of the SVM algorithm [21] as implemented in the LIBSVM [13] software library.

#### Naïve-Bayes classifier

The original FEATURE program (FEATURE-NB) used Naïve-Bayes classifier models. The Naïve-Bayes learning algorithm estimates class-conditional probability density functions for each class 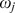 by assuming independence of individual features:
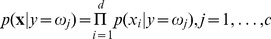
(4)where 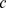 is the number of classes and 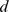 the number of features (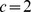 and 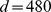 in this work). Class-conditional posterior probability estimates are derived by combining the density functions and class probabilities using Bayes theorem:



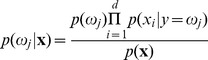
(5)The decision function assigns a given feature vector 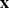 to the class with the maximum estimated posterior probability:
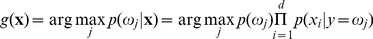
(6)


We treated 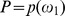 as a tunable parameter. We approximated 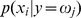 using the training data and dividing the observed values into a histogram of five bins [8].

### Predictive Model Selection and Performance Estimation

Performance evaluation of FEATURE included selection of the best classification model for each site. The different models were built by varying the top-level parameter 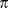 (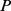 for Naïve Bayes, 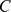 for SVM). We performed model selection by comparing cross-validation estimates of performance for the different models, and selecting as the best model the one producing the minimum number of misclassifications. For each model corresponding to a different value of the top-level parameter, we also recorded the estimated class-conditional posterior probabilities for each sample.

Once the best model was chosen for each functional site, we calculated precision and recall using the recorded class-conditional probabilities. This required setting a decision threshold to achieve the stated goal of 99% precision. In a finite-sample scenario, it is not possible to achieve the exactly specified value of precision; we used the closest achievable value. The actual achieved precision values are reported in the Results section.

The model selection process used the positive and negative feature vectors and performed a grid search of user-tunable parameters (cost C for FEATURE-SVM, prior probability of the positive class P for FEATURE-NB) yielding the best model. The search amounted to selecting the model parameters which produced the highest recall given a precision, estimated using cross-validation as described below. The optimization of the parameters 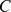 and 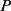 was conducted over a pre-defined set of values. For each value, we performed the five-fold cross-validation estimation of the performance of the classifier. Based on published guidelines [22] and the authors’ experience, we used the following set of SVM cost grid values on the 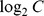 scale: 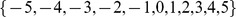. Classifiers built using Naïve Bayes utilized a previously published [8] grid of values 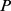 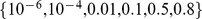.

By necessity, the number of positive examples was significantly smaller than the number of negative examples. To ameliorate the impact of the highly unbalanced classes, we used stratification by class label, whereby each cross-validation fold had approximately the same proportion of positive and negative examples as the overall training set.

The cross-validation algorithm for a given top-level parameter 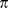 is defined in Algorithm 1 box. The number of folds 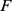 was set to five.

**Algorithm 1 The cross-validation approach for generating sample predictions for a given functional site and associated training dataset *D***

**Require:** dataset *D*, subsets *D*_1,_ *D*_2_ … *D_F_*, parameters *π*
 
**for** *i* = 1 … *F* **do**
  
*Learning Set_i_* = *D*\*D_i_*
  
*Cross Validation Set_i_* = *D_i_*
  
*Model_i_* = *Train*(*Learning Set_i_*, *π*)
  
*Predictions_i_* = *Predict*(*Model_i_*, *Cross Validation Set_i_*)
 
**end for**
 
*Predictions =* 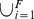 *Predictions_i_*

This approach highlights the following question related to estimation of model performance in the cross-validation setting. 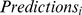 is the set of probability estimates for examples in subset 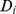. The union of all 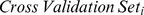 sets contains the entire training set, so the above procedure generates probability estimates 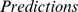 for all training set examples. In principle, this is the required input data for estimating classifier performance. However, the individual prediction sets 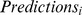 were generated by 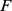 different models, and are therefore not directly comparable. To the best of our knowledge, there is no consensus in the machine learning community on how to produce aggregate measures in this scenario [23]. We took the approach of treating all 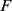 cross-validation iterations as a single continuous experiment, although other approaches may be sensible.

The 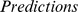 probability estimates were used to calculate all statistics reported in the Results section.

### Comparison of FEATURE with Pfam

The key challenge in comparing different annotators is matching their respective functional site assignments. In our case, FEATURE produces functional sites predictions as defined by PROSITE, because that is where the “truth” labels for FEATURE models are derived from. Pfam has its own nomenclature of functional sites, creating the challenge of comparing predictions for the two methods. To resolve this and estimate Pfam predictive performance on a scale comparable to FEATURE, we developed a protocol utilizing InterPro [24], a resource which unites diverse protein annotation databases, including PROSITE and Pfam. The protocol consisted of the following steps for each of the 20 protein models we analyzed:

- Record PROSITE accession number for the functional site. FEATURE predictive models are functional site predictors based on PROSITE patterns, therefore by definition each has a PROSITE accession number.
- Record all Pfam annotations that are co-located with PROSITE annotations (identified by the PROSITE accession number) in InterPro. To increase confidence in the mapping, we only used InterPro mapping entries for which the corresponding protein exists in SWISSPROT [25].
  As an example, PROSITE ASP_PROTEASE (PS00141) maps to two Pfam domains: Eukaryotic aspartyl protease (PF00026) and Retroviral aspartyl protease (PF00077). The mapping of all 20 protein models is listed in Table 3.
- Generate Pfam predictions (domains) using the amino-acid sequence data for the positive and negative examples as input.
- Calculate Pfam precision and recall using the PROSITE-to-Pfam mapping. The confusion matrix was generated using the following logic:

 – For positive examples, if any of the Pfam predictions matched PROSITE as per Table 3 mapping, we considered the prediction a True Positive; if none of the Pfam predictions matched PROSITE, we considered the prediction a False Negative.
   As an example, consider an ASP_PROTEASE positive example. If Pfam prediction for the example contained Eukaryotic aspartyl protease (PF00026) or Retroviral aspartyl protease (PF00077), it was considered a True Positive.
 – For negative examples, if any of the Pfam predictions matched PROSITE, we considered the prediction a False Positive; if none of the Pfam predictions matched PROSITE, we considered the prediction a True Negative.

**Table 3:** PROSITE/Pfam mapping of the functional families.

PROSITE	Pfam/InterPro
**ADH_SHORT**	ADH_SHORT, NAD dependent epimerase/dehydratase
**ALPHA_CA_1**	Eukaryotic-type carbonic anhydrase
**ASP_PROTEASE**	Retroviral aspartyl protease, Eukaryotic aspartyl protease
**ATPASE_ALPHA_BETA**	ATP synthase alpha/beta family
**CARBOXYLESTERASE_B_1**	Carboxylesterase family, Alpha/beta hydrolase fold
**CYTOCHROME_P450**	Cytochrome P450
**EF_HAND**	EF-hand, EF, Dockerin, Secreted
**EGF_1**	Laminin EGF-like, hEGF, EGF-like domain, Ca-binding EGF
**IG_MHC**	IG C1 Set, IG V Set
**INSULIN**	Insulin/IGF/Relaxin family, Nematode insulin-related peptide beta type
**LACTALBUMIN_LYSOZYME**	C-type lysozyme/alpha-lactalbumin family
**LECTIN_LEGUME_BETA**	Lectin_leg*β*
**PA2_HIS**	Phospholip_A2_1, Phospholipase A2, PLA2G12
**PROTEIN_KINASE_ST**	Protein kinase domain, Protein tyrosine kinase
**PROTEIN_KINASE_TYR**	Protein tyrosine kinase, Protein kinase domain, RIO1 family, Lipopolysaccharide kinase (Kdo/WaaP) family
**RNASE_PANCREATIC**	Pancreatic ribonuclease
**SOD_CU_ZN_1**	Copper/zinc superoxide dismutase
**TRYPSIN_HIS**	TRYPSIN
**TRYPSIN_SER**	TRYPSIN, Immunoglobulin A1 Protease
**ZINC_PROTEASE**	NO MATCH FOUND

Column PROSITE lists functional families used to evaluate performance of FEATURE. Column Pfam/InterPro lists corresponding Pfam families used to compare performance of FEATURE and Pfam. The correspondence was established through the InterPro database as described in the text.

One of the functional sites (ZINC_PROTEASE) did not have a matching InterPro entry and therefore was not used in Pfam analyses because there was no pre-specified way to compare the FEATURE and Pfam predictions for that site.

This protocol does not provide an opportunity to control the precision/recall trade-off. Therefore the Pfam results were reported at whatever precision level was reached with Pfam.

### Computations

Training and evaluation of SVM machine learning on all PROSITE v20.80 functional classes demanded large-scale parallel computation. Feature extraction, parameter optimization and cross-validation takes 4–8 hours on an Intel Xeon 3400-series processor for a typical SVM predictive model, the most computationally demanding of the three methods considered here. To meet this challenge, all computations were performed using Amazon Elastic Cloud Computing (EC2) services with MIT StarCluster software [26]. Amazon EC2 provides virtual machines (VMs) for scalable cost-efficient computation. MIT StarCluster organizes these VMs into a dynamically scalable Beowulf cluster with parallel computing tools such as MPI and Open Grid Scheduler.

## Results

We extracted positive and negative examples using the protocol described in the Materials section. The resulting numbers of examples, given in Table 4, provided for narrow 95% confidence intervals of the estimated performance parameters and robust conclusions regarding the methods’ performances.

**Table 4:** Number of positive and negative examples for each functional site.

PROSITE Functional Family	Positive examples	Negative examples
**ADH_SHORT**	373	50130
**ALPHA_CA_1**	422	50000
**ASP_PROTEASE**	1585	48445
**ATPASE_ALPHA_BETA**	369	50000
**CARBOXYLESTERASE_B_1**	345	50000
**CYTOCHROME_P450**	393	50000
**EF_HAND**	1811	48435
**EGF_1**	138	50058
**IG_MHC**	2017	49098
**INSULIN**	826	49078
**LACTALBUMIN_LYSOZYME**	649	50024
**LECTIN_LEGUME_BETA**	459	50007
**PA2_HIS**	382	50003
**PROTEIN_KINASE_ST**	1096	50000
**PROTEIN_KINASE_TYR**	275	50010
**RNASE_PANCREATIC**	384	50000
**SOD_CU_ZN_1**	392	47506
**TRYPSIN_HIS**	446	47490
**TRYPSIN_SER**	317	48034
**ZINC_PROTEASE**	649	50028

Due to finite training set size, precision could not be set exactly at 99%. We used the closest achievable value for FEATURE-SVM and FEATURE-NB, as reported in Table 5. For Pfam, no precision tuning was possible, but with the exception of ADH_SHORT and KINASE_TYR it also provided precision exceeding 99% (for ADH_SHORT and KINASE_TYR the Pfam precision values were 98% and 96%, respectively).

**Table 5:** Precision and recall values achieved by different classifiers.

Functional Family	SVM P/R		NB P/R		Pfam P/R	
**ADH_SHORT**	98.9	98.4	98.9	97.3	97.9	37.3
**ALPHA_CA_1**	99.1	100.0	99.1	100.0	100.0	93.8
**ASP_PROTEASE**	99.0	100.0	99.3	95.8	100.0	57.2
**ATPASE_ALPHA_BETA**	98.9	99.7	99.0	81.3	100.0	22.2
**CARBOXYLESTERASE_B_1**	99.1	100.0	99.1	100.0	100.0	67.0
**CYTOCHROME_P450**	99.0	100.0	99.0	99.7	100.0	57.8
**EF_HAND**	99.0	87.9	99.0	64.0	99.3	58.7
**EGF_1**	99.0	71.7	100.0	11.6	100.0	74.6
**IG_MHC**	99.0	90.5	99.0	73.0	100.0	65.4
**INSULIN**	99.0	94.3	98.8	60.4	100.0	42.9
**LACTALBUMIN_LYSOZYME**	99.1	99.8	99.1	99.8	100.0	86.0
**LECTIN_LEGUME_BETA**	98.9	99.6	98.9	99.1	100.0	36.2
**PA2_HIS**	99.0	100.0	99.7	95.0	100.0	61.0
**PROTEIN_KINASE_ST**	99.0	95.3	99.1	72.6	100.0	67.3
**PROTEIN_KINASE_TYR**	99.2	92.0	99.4	63.6	96.3	76.4
**RNASE_PANCREATIC**	99.1	87.8	99.1	82.0	100.0	76.0
**SOD_CU_ZN_1**	99.0	100.0	99.0	99.2	100.0	30.6
**TRYPSIN_HIS**	99.0	93.0	99.0	91.7	100.0	84.8
**TRYPSIN_SER**	99.3	87.7	99.2	81.7	99.2	76.7
**ZINC_PROTEASE**	99.1	99.1	99.5	94.8		

The values are given in percents. SVM: FEATURE-SVM; NB: FEATURE-NB; P/R: Precision/Recall. PROSITE-Pfam mapping was not available for ZINC_PROSITE, and thus no Pfam results were obtained.

Overall, FEATURE-SVM clearly surpassed Pfam and FEATURE-NB in terms of recall at approximately 99% precision (Fig. 1, Table 5 and Figures S1–S20 in File S1). For 18 out of the 20 functional sites, the difference between the FEATURE-SVM recall rate and that of Pfam was between 6% and 78%. All differences were statistically significant with 95% confidence. For one site (EGF_1), Pfam recall rate was slightly higher than FEATURE-SVM (75% vs. 72%), though the difference was not statistically significant. The Pfam result for ZINC_PROTEASE was not available because InterPro did not have a corresponding Pfam match.

**Figure 1:**
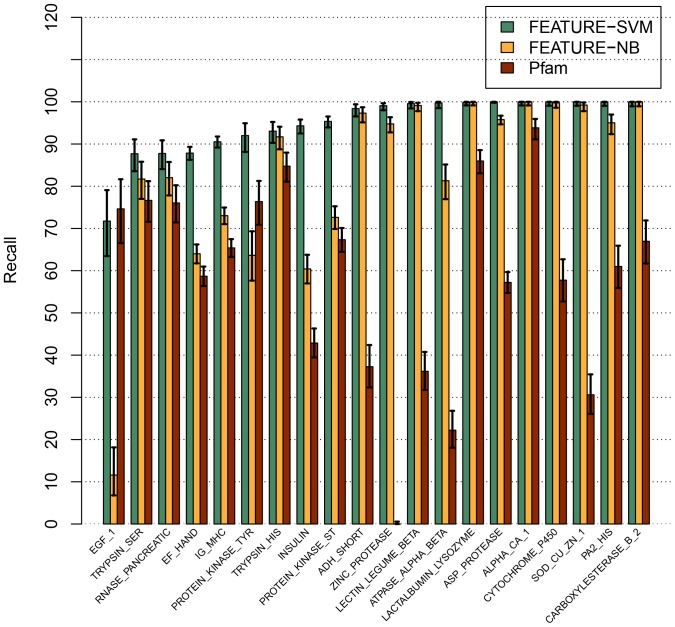
Performance comparison of FEATURE-SVM, original FEATURE (FEATURE-NB) and Pfam. y-axis is recall value at approximately 99% precision. Vertical lines within bars indicate 95% confidence intervals. Pfam result for ZINC_PROTEASE was not available because the InterPro database, which was used to map site names, does not have a mapping record for this functional site. The functional sites are sorted by increasing recall value of FEATURE-SVM.

FEATURE-SVM was superior to FEATURE-NB for 16 sites by between 1% and 60%. In ten out of the 16 comparisons the difference was statistically significant with 95% confidence. For LACTALBUMIN_LYSOZYME, ALPHA_CA_1, CYTOCHROME_P450 and CARBOXYLESTERASE_B_2, both FEATURE-SVM and FEATURE-NB achieved 100% recall. In summary, for the 19 sites for which all three methods yielded a result, the mean recall rates were 95% (FEATURE-SVM), 83% (FEATURE-NB) and 59% (Pfam).

## Discussion

We sought to develop a system for identifying functional sites in protein structures for an important use case scenario. Specifically, our goal was to develop an annotator that achieves acceptable levels of recall at 99% precision. We found that the combination of FEATURE and Support Vector Machine classifier delivered high recall (exceeding 70% in all of the cases studied, and averaging 95% over 20 functional sites) at the specified level of precision. This met our goals and thus we are able to provide a useful new tool (through the WebFEATURE service) for researchers in this domain, especially given the magnitude of the absolute and relative performance gain (95% recall vs. 83% for FEATURE-NB and 59% for Pfam).

We observed that the Support Vector Machine classifier delivered better classification accuracy than Naïve Bayes (95% recall vs. 83% for the FEATURE-NB averaged over all 20 functional sites). This is consistent with observations in many other application domains (for example cancer diagnostics [27]) and further confirms the power of this classification model.

The FEATURE-SVM annotator is purely predictive and does not explain to what extent individual microenvironment attributes contribute to the functionality of the predicted site. This behavior is a consequence of our focus on maximizing accuracy (i.e., precision/recall). It is consistent with recent findings in causal inference [28] that demonstrate that ranking of features for classification may have no explanatory utility.

When evaluating annotators for our use case scenario (i.e., prediction of function in a solved structure followed by experimental confirmation), it is important to note that the FEATURE-based tools point to exact atomic location of the functional site, unlike Pfam, which reports a (sometimes long) sequence segment corresponding to a functional domain.

We performed exhaustive analysis of 20 functional sites, which is a small fraction of the potentially useful sites (the number offered through the WebFEATURE service is over 600). Nevertheless, we argue that our main conclusion of high utility of the FEATURE-SVM annotator is likely to apply to the general population of sites for the following reasons:

- The 20 sites were chosen *a priori*, before any analyses, and then frozen, which makes for an unbiased sample.
- Given the magnitude of the estimated recall (95%), even if the estimate is biased, the large-sample estimate is still likely to be in the very useful range.

We developed a protocol for measuring Pfam performance in a way that is comparable to FEATURE. There is no single best way to do this since the mapping of functional sites from Pfam to FEATURE involves a degree of expert judgment. We argue that our protocol does not favor FEATURE for the following reason. Pfam may predict multiple domains for a given input sequence. If any of the predicted domains matches PROSITE per the established mapping, we consider the prediction to be a True Positive. Therefore we believe that the FEATURE performance relative to Pfam observed in practice is likely to be as good as reported here or better.

The choice of Pfam as the primary 1D function prediction method for the comparison was somewhat arbitrary. It is based on the fact that Pfam is a well-recognized tool, and that it represents a class of sequence-based methods with similar performance. Thus our comparative results should be representative of the expected performance gap between FEATURE-SVM and 1D methods.

We performed extensive and rigorous evaluation of the methods we used, with over 50,000 training examples for each functional class and extensive grid-search of the user-tunable parameters using cross-validation. To the best of our knowledge, no other annotator has been evaluated in a comparable manner.

End user of a functional annotator system would benefit from a rigorous performance comparison of competing state-of-the-art structural methods. However, we are not aware of another predictive algorithm which has been evaluated in the way performed in this paper, therefore direct comparison with our work is not possible. Furthermore, a key requirement for the comparison of different predictor outputs is translation to a common “language” of functional sites. As illustrated in our FEATURE - Pfam comparison, this requires extensive automation and human judgment, and is beyond scope of the present report. We leave a comparison of FEATURE to other structural methods for future research.

## Conclusions

The combination of FEATURE properties and Support Vector Machine classifier predicts precise location of functional sites in unannotated protein structures with 99% precision and high recall rates (exceeding 70% in all of the cases studied, and averaging 95%). As a result, the WebFEATURE service which implements the FEATURE predictive models allows users to confidently pursue laboratory confirmation of the predicted protein function. Additionally, our findings suggest that bioinformaticians interested in predictive modeling of protein activity should consider Support Vector Machine classifiers for the most accurate results.

## Supporting Information

File S1 **This file contains Figures S1–S20, which are recall vs. precision graphs for the 20 models analyzed in the paper.** Figure S1, Recall vs. Precision: ADH_SHORT. Figure S2, Recall vs. Precision: Alpha_CA_1. Figure S3, Recall vs. Precision: ASP_PROTEASE. Figure S4, Recall vs. Precision: ATPASE_ALPHA_BETA. Figure S5, Recall vs. Precision: CARBOXYLESTERASE_B_2. Figure S6, Recall vs. Precision: CYTOCHROME_P450. Figure S7, Recall vs. Precision: EF_HAND. Figure S8, Recall vs. Precision: EGF_1. Figure S9, Recall vs. Precision: IG_MHG. Figure S10, Recall vs. Precision: INSULIN. Figure S11, Recall vs. Precision: LACTALBUMIN_LYSOZYME. Figure S12, Recall vs. Precision: LECTIN_LEGUME_BETA. Figure S13, Recall vs. Precision: PA2_HIS. Figure S14, Recall vs. Precision: PROTEIN_KINASE_ST. Figure S15, Recall vs. Precision: PROTEIN_KINASE_TYR. Figure S16, Recall vs. Precision: RNASE_PANCREATIC. Figure S17, Recall vs. Precision: SOD_CU_ZN_1. Figure S18, Recall vs. Precision: TRYPSIN_HIS. Figure S19, Recall vs. Precision: TRYPSIN _SER. Figure S20, Recall vs. Precision: ZINC_PROTEASE.
(ZIP)
