# Supplementary figures and images for: High Precision Prediction of Functional Sites in Protein Structures
Source: PLoS One. 2014 Mar 14;9(3):e91240. doi: 10.1371/journal.pone.0091240 (PMC3954699; doi:10.1371/journal.pone.0091240)

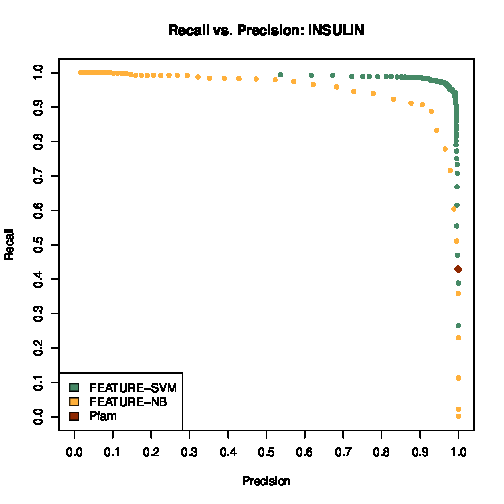

Supplement: File S1 — This file contains Figures S1–S20, which are recall vs. precision graphs for the 20 models analyzed in the paper. Figure S1, Recall vs. Precision: ADH_SHORT. Figure S2, Recall vs. Precision: Alpha_CA_1. Figure S3, Recall vs. Precision: ASP_PROTEASE. Figure S4, Recall vs. Precision: ATPASE_ALPHA_BETA. Figure S5, Recall vs. Precision: CARBOXYLESTERASE_B_2. Figure S6, Recall vs. Precision: CYTOCHROME_P450. Figure S7, Recall vs. Precision: EF_HAND. Figure S8, Recall vs. Precision: EGF_1. Figure S9, Recall vs. Precision: IG_MHG. Figure S10, Recall vs. Precision: INSULIN. Figure S11, Recall vs. Precision: LACTALBUMIN_LYSOZYME. Figure S12, Recall vs. Precision: LECTIN_LEGUME_BETA. Figure S13, Recall vs. Precision: PA2_HIS. Figure S14, Recall vs. Precision: PROTEIN_KINASE_ST. Figure S15, Recall vs. Precision: PROTEIN_KINASE_TYR. Figure S16, Recall vs. Precision: RNASE_PANCREATIC. Figure S17, Recall vs. Precision: SOD_CU_ZN_1. Figure S18, Recall vs. Precision: TRYPSIN_HIS. Figure S19, Recall vs. Precision: TRYPSIN _SER. Figure S20, Recall vs. Precision: ZINC_PROTEASE. (ZIP) [file pone.0091240.s001.zip › Figure_S10_INSULIN.tiff]

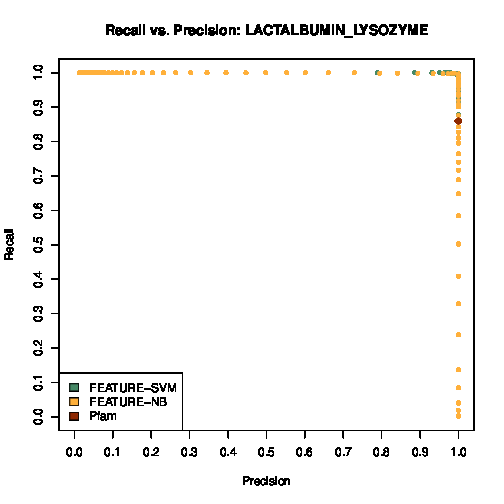

Supplement: File S1 — This file contains Figures S1–S20, which are recall vs. precision graphs for the 20 models analyzed in the paper. Figure S1, Recall vs. Precision: ADH_SHORT. Figure S2, Recall vs. Precision: Alpha_CA_1. Figure S3, Recall vs. Precision: ASP_PROTEASE. Figure S4, Recall vs. Precision: ATPASE_ALPHA_BETA. Figure S5, Recall vs. Precision: CARBOXYLESTERASE_B_2. Figure S6, Recall vs. Precision: CYTOCHROME_P450. Figure S7, Recall vs. Precision: EF_HAND. Figure S8, Recall vs. Precision: EGF_1. Figure S9, Recall vs. Precision: IG_MHG. Figure S10, Recall vs. Precision: INSULIN. Figure S11, Recall vs. Precision: LACTALBUMIN_LYSOZYME. Figure S12, Recall vs. Precision: LECTIN_LEGUME_BETA. Figure S13, Recall vs. Precision: PA2_HIS. Figure S14, Recall vs. Precision: PROTEIN_KINASE_ST. Figure S15, Recall vs. Precision: PROTEIN_KINASE_TYR. Figure S16, Recall vs. Precision: RNASE_PANCREATIC. Figure S17, Recall vs. Precision: SOD_CU_ZN_1. Figure S18, Recall vs. Precision: TRYPSIN_HIS. Figure S19, Recall vs. Precision: TRYPSIN _SER. Figure S20, Recall vs. Precision: ZINC_PROTEASE. (ZIP) [file pone.0091240.s001.zip › Figure_S11_LACTALBUMIN_LYSOZYME.tiff]

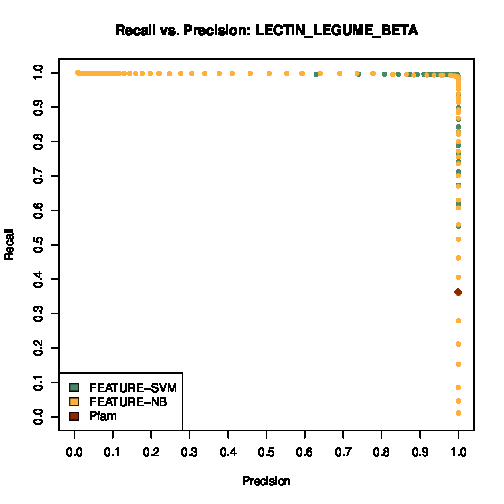

Supplement: File S1 — This file contains Figures S1–S20, which are recall vs. precision graphs for the 20 models analyzed in the paper. Figure S1, Recall vs. Precision: ADH_SHORT. Figure S2, Recall vs. Precision: Alpha_CA_1. Figure S3, Recall vs. Precision: ASP_PROTEASE. Figure S4, Recall vs. Precision: ATPASE_ALPHA_BETA. Figure S5, Recall vs. Precision: CARBOXYLESTERASE_B_2. Figure S6, Recall vs. Precision: CYTOCHROME_P450. Figure S7, Recall vs. Precision: EF_HAND. Figure S8, Recall vs. Precision: EGF_1. Figure S9, Recall vs. Precision: IG_MHG. Figure S10, Recall vs. Precision: INSULIN. Figure S11, Recall vs. Precision: LACTALBUMIN_LYSOZYME. Figure S12, Recall vs. Precision: LECTIN_LEGUME_BETA. Figure S13, Recall vs. Precision: PA2_HIS. Figure S14, Recall vs. Precision: PROTEIN_KINASE_ST. Figure S15, Recall vs. Precision: PROTEIN_KINASE_TYR. Figure S16, Recall vs. Precision: RNASE_PANCREATIC. Figure S17, Recall vs. Precision: SOD_CU_ZN_1. Figure S18, Recall vs. Precision: TRYPSIN_HIS. Figure S19, Recall vs. Precision: TRYPSIN _SER. Figure S20, Recall vs. Precision: ZINC_PROTEASE. (ZIP) [file pone.0091240.s001.zip › Figure_S12_LECTIN_LEGUME_BETA.tiff]

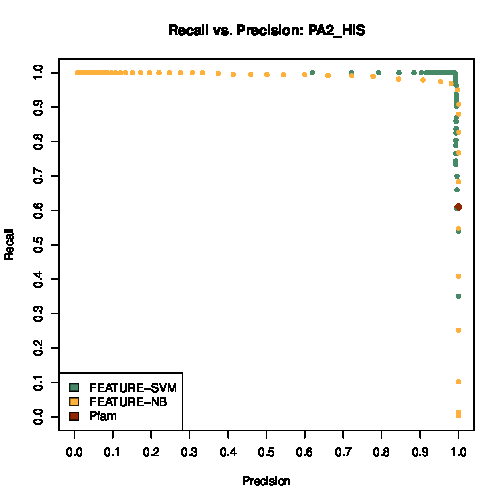

Supplement: File S1 — This file contains Figures S1–S20, which are recall vs. precision graphs for the 20 models analyzed in the paper. Figure S1, Recall vs. Precision: ADH_SHORT. Figure S2, Recall vs. Precision: Alpha_CA_1. Figure S3, Recall vs. Precision: ASP_PROTEASE. Figure S4, Recall vs. Precision: ATPASE_ALPHA_BETA. Figure S5, Recall vs. Precision: CARBOXYLESTERASE_B_2. Figure S6, Recall vs. Precision: CYTOCHROME_P450. Figure S7, Recall vs. Precision: EF_HAND. Figure S8, Recall vs. Precision: EGF_1. Figure S9, Recall vs. Precision: IG_MHG. Figure S10, Recall vs. Precision: INSULIN. Figure S11, Recall vs. Precision: LACTALBUMIN_LYSOZYME. Figure S12, Recall vs. Precision: LECTIN_LEGUME_BETA. Figure S13, Recall vs. Precision: PA2_HIS. Figure S14, Recall vs. Precision: PROTEIN_KINASE_ST. Figure S15, Recall vs. Precision: PROTEIN_KINASE_TYR. Figure S16, Recall vs. Precision: RNASE_PANCREATIC. Figure S17, Recall vs. Precision: SOD_CU_ZN_1. Figure S18, Recall vs. Precision: TRYPSIN_HIS. Figure S19, Recall vs. Precision: TRYPSIN _SER. Figure S20, Recall vs. Precision: ZINC_PROTEASE. (ZIP) [file pone.0091240.s001.zip › Figure_S13_PA2_HIS.tiff]

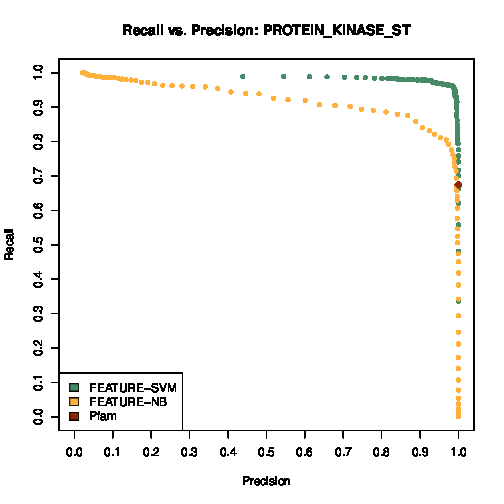

Supplement: File S1 — This file contains Figures S1–S20, which are recall vs. precision graphs for the 20 models analyzed in the paper. Figure S1, Recall vs. Precision: ADH_SHORT. Figure S2, Recall vs. Precision: Alpha_CA_1. Figure S3, Recall vs. Precision: ASP_PROTEASE. Figure S4, Recall vs. Precision: ATPASE_ALPHA_BETA. Figure S5, Recall vs. Precision: CARBOXYLESTERASE_B_2. Figure S6, Recall vs. Precision: CYTOCHROME_P450. Figure S7, Recall vs. Precision: EF_HAND. Figure S8, Recall vs. Precision: EGF_1. Figure S9, Recall vs. Precision: IG_MHG. Figure S10, Recall vs. Precision: INSULIN. Figure S11, Recall vs. Precision: LACTALBUMIN_LYSOZYME. Figure S12, Recall vs. Precision: LECTIN_LEGUME_BETA. Figure S13, Recall vs. Precision: PA2_HIS. Figure S14, Recall vs. Precision: PROTEIN_KINASE_ST. Figure S15, Recall vs. Precision: PROTEIN_KINASE_TYR. Figure S16, Recall vs. Precision: RNASE_PANCREATIC. Figure S17, Recall vs. Precision: SOD_CU_ZN_1. Figure S18, Recall vs. Precision: TRYPSIN_HIS. Figure S19, Recall vs. Precision: TRYPSIN _SER. Figure S20, Recall vs. Precision: ZINC_PROTEASE. (ZIP) [file pone.0091240.s001.zip › Figure_S14_PROTEIN_KINASE_ST.tiff]

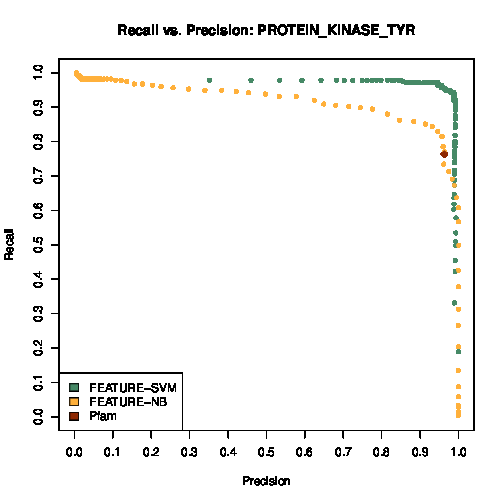

Supplement: File S1 — This file contains Figures S1–S20, which are recall vs. precision graphs for the 20 models analyzed in the paper. Figure S1, Recall vs. Precision: ADH_SHORT. Figure S2, Recall vs. Precision: Alpha_CA_1. Figure S3, Recall vs. Precision: ASP_PROTEASE. Figure S4, Recall vs. Precision: ATPASE_ALPHA_BETA. Figure S5, Recall vs. Precision: CARBOXYLESTERASE_B_2. Figure S6, Recall vs. Precision: CYTOCHROME_P450. Figure S7, Recall vs. Precision: EF_HAND. Figure S8, Recall vs. Precision: EGF_1. Figure S9, Recall vs. Precision: IG_MHG. Figure S10, Recall vs. Precision: INSULIN. Figure S11, Recall vs. Precision: LACTALBUMIN_LYSOZYME. Figure S12, Recall vs. Precision: LECTIN_LEGUME_BETA. Figure S13, Recall vs. Precision: PA2_HIS. Figure S14, Recall vs. Precision: PROTEIN_KINASE_ST. Figure S15, Recall vs. Precision: PROTEIN_KINASE_TYR. Figure S16, Recall vs. Precision: RNASE_PANCREATIC. Figure S17, Recall vs. Precision: SOD_CU_ZN_1. Figure S18, Recall vs. Precision: TRYPSIN_HIS. Figure S19, Recall vs. Precision: TRYPSIN _SER. Figure S20, Recall vs. Precision: ZINC_PROTEASE. (ZIP) [file pone.0091240.s001.zip › Figure_S15_PROTEIN_KINASE_TYR.tiff]

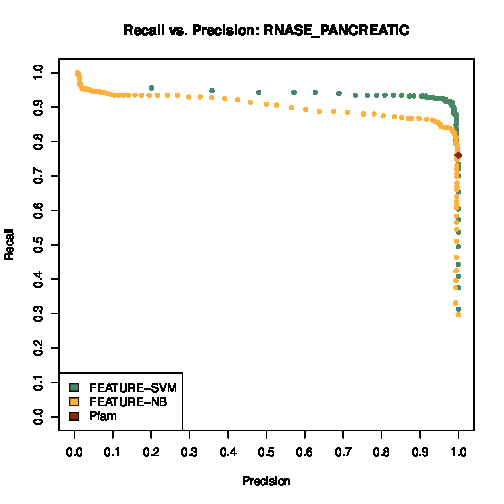

Supplement: File S1 — This file contains Figures S1–S20, which are recall vs. precision graphs for the 20 models analyzed in the paper. Figure S1, Recall vs. Precision: ADH_SHORT. Figure S2, Recall vs. Precision: Alpha_CA_1. Figure S3, Recall vs. Precision: ASP_PROTEASE. Figure S4, Recall vs. Precision: ATPASE_ALPHA_BETA. Figure S5, Recall vs. Precision: CARBOXYLESTERASE_B_2. Figure S6, Recall vs. Precision: CYTOCHROME_P450. Figure S7, Recall vs. Precision: EF_HAND. Figure S8, Recall vs. Precision: EGF_1. Figure S9, Recall vs. Precision: IG_MHG. Figure S10, Recall vs. Precision: INSULIN. Figure S11, Recall vs. Precision: LACTALBUMIN_LYSOZYME. Figure S12, Recall vs. Precision: LECTIN_LEGUME_BETA. Figure S13, Recall vs. Precision: PA2_HIS. Figure S14, Recall vs. Precision: PROTEIN_KINASE_ST. Figure S15, Recall vs. Precision: PROTEIN_KINASE_TYR. Figure S16, Recall vs. Precision: RNASE_PANCREATIC. Figure S17, Recall vs. Precision: SOD_CU_ZN_1. Figure S18, Recall vs. Precision: TRYPSIN_HIS. Figure S19, Recall vs. Precision: TRYPSIN _SER. Figure S20, Recall vs. Precision: ZINC_PROTEASE. (ZIP) [file pone.0091240.s001.zip › Figure_S16_RNASE_PANCREATIC.tiff]

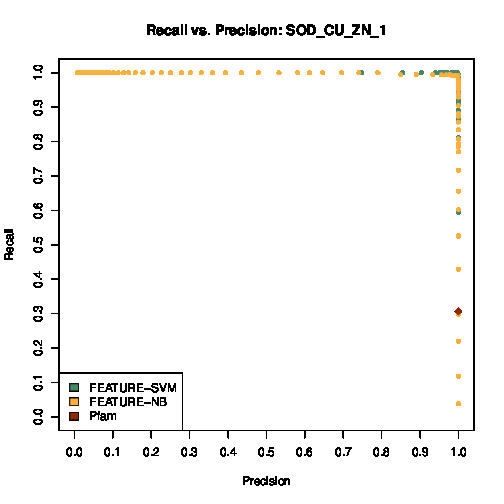

Supplement: File S1 — This file contains Figures S1–S20, which are recall vs. precision graphs for the 20 models analyzed in the paper. Figure S1, Recall vs. Precision: ADH_SHORT. Figure S2, Recall vs. Precision: Alpha_CA_1. Figure S3, Recall vs. Precision: ASP_PROTEASE. Figure S4, Recall vs. Precision: ATPASE_ALPHA_BETA. Figure S5, Recall vs. Precision: CARBOXYLESTERASE_B_2. Figure S6, Recall vs. Precision: CYTOCHROME_P450. Figure S7, Recall vs. Precision: EF_HAND. Figure S8, Recall vs. Precision: EGF_1. Figure S9, Recall vs. Precision: IG_MHG. Figure S10, Recall vs. Precision: INSULIN. Figure S11, Recall vs. Precision: LACTALBUMIN_LYSOZYME. Figure S12, Recall vs. Precision: LECTIN_LEGUME_BETA. Figure S13, Recall vs. Precision: PA2_HIS. Figure S14, Recall vs. Precision: PROTEIN_KINASE_ST. Figure S15, Recall vs. Precision: PROTEIN_KINASE_TYR. Figure S16, Recall vs. Precision: RNASE_PANCREATIC. Figure S17, Recall vs. Precision: SOD_CU_ZN_1. Figure S18, Recall vs. Precision: TRYPSIN_HIS. Figure S19, Recall vs. Precision: TRYPSIN _SER. Figure S20, Recall vs. Precision: ZINC_PROTEASE. (ZIP) [file pone.0091240.s001.zip › Figure_S17_SOD_CU_ZN_1.tiff]

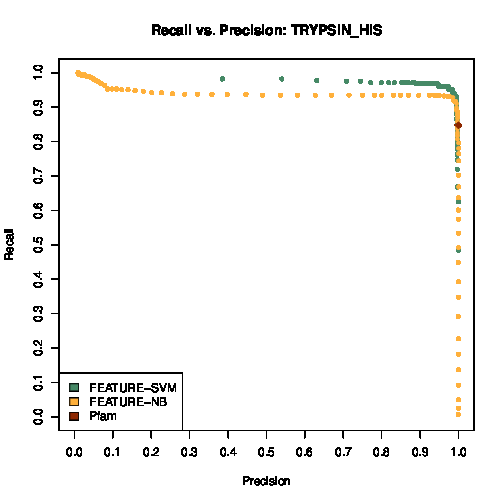

Supplement: File S1 — This file contains Figures S1–S20, which are recall vs. precision graphs for the 20 models analyzed in the paper. Figure S1, Recall vs. Precision: ADH_SHORT. Figure S2, Recall vs. Precision: Alpha_CA_1. Figure S3, Recall vs. Precision: ASP_PROTEASE. Figure S4, Recall vs. Precision: ATPASE_ALPHA_BETA. Figure S5, Recall vs. Precision: CARBOXYLESTERASE_B_2. Figure S6, Recall vs. Precision: CYTOCHROME_P450. Figure S7, Recall vs. Precision: EF_HAND. Figure S8, Recall vs. Precision: EGF_1. Figure S9, Recall vs. Precision: IG_MHG. Figure S10, Recall vs. Precision: INSULIN. Figure S11, Recall vs. Precision: LACTALBUMIN_LYSOZYME. Figure S12, Recall vs. Precision: LECTIN_LEGUME_BETA. Figure S13, Recall vs. Precision: PA2_HIS. Figure S14, Recall vs. Precision: PROTEIN_KINASE_ST. Figure S15, Recall vs. Precision: PROTEIN_KINASE_TYR. Figure S16, Recall vs. Precision: RNASE_PANCREATIC. Figure S17, Recall vs. Precision: SOD_CU_ZN_1. Figure S18, Recall vs. Precision: TRYPSIN_HIS. Figure S19, Recall vs. Precision: TRYPSIN _SER. Figure S20, Recall vs. Precision: ZINC_PROTEASE. (ZIP) [file pone.0091240.s001.zip › Figure_S18_TRYPSIN_HIS.tiff]

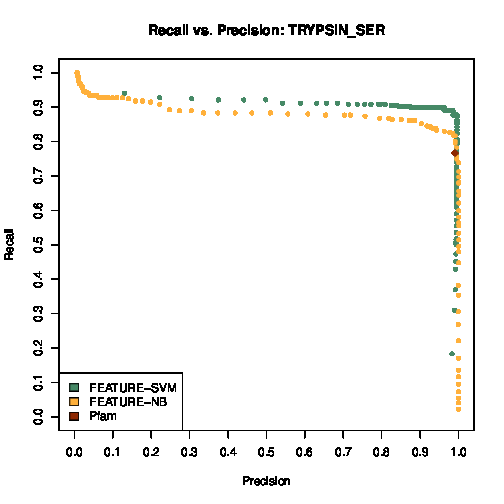

Supplement: File S1 — This file contains Figures S1–S20, which are recall vs. precision graphs for the 20 models analyzed in the paper. Figure S1, Recall vs. Precision: ADH_SHORT. Figure S2, Recall vs. Precision: Alpha_CA_1. Figure S3, Recall vs. Precision: ASP_PROTEASE. Figure S4, Recall vs. Precision: ATPASE_ALPHA_BETA. Figure S5, Recall vs. Precision: CARBOXYLESTERASE_B_2. Figure S6, Recall vs. Precision: CYTOCHROME_P450. Figure S7, Recall vs. Precision: EF_HAND. Figure S8, Recall vs. Precision: EGF_1. Figure S9, Recall vs. Precision: IG_MHG. Figure S10, Recall vs. Precision: INSULIN. Figure S11, Recall vs. Precision: LACTALBUMIN_LYSOZYME. Figure S12, Recall vs. Precision: LECTIN_LEGUME_BETA. Figure S13, Recall vs. Precision: PA2_HIS. Figure S14, Recall vs. Precision: PROTEIN_KINASE_ST. Figure S15, Recall vs. Precision: PROTEIN_KINASE_TYR. Figure S16, Recall vs. Precision: RNASE_PANCREATIC. Figure S17, Recall vs. Precision: SOD_CU_ZN_1. Figure S18, Recall vs. Precision: TRYPSIN_HIS. Figure S19, Recall vs. Precision: TRYPSIN _SER. Figure S20, Recall vs. Precision: ZINC_PROTEASE. (ZIP) [file pone.0091240.s001.zip › Figure_S19_TRYPSIN_SER.tiff]

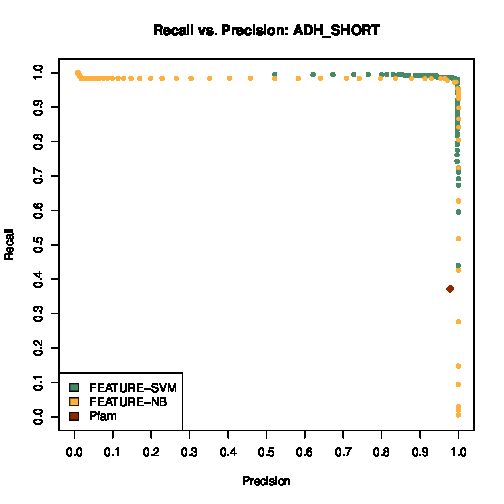

Supplement: File S1 — This file contains Figures S1–S20, which are recall vs. precision graphs for the 20 models analyzed in the paper. Figure S1, Recall vs. Precision: ADH_SHORT. Figure S2, Recall vs. Precision: Alpha_CA_1. Figure S3, Recall vs. Precision: ASP_PROTEASE. Figure S4, Recall vs. Precision: ATPASE_ALPHA_BETA. Figure S5, Recall vs. Precision: CARBOXYLESTERASE_B_2. Figure S6, Recall vs. Precision: CYTOCHROME_P450. Figure S7, Recall vs. Precision: EF_HAND. Figure S8, Recall vs. Precision: EGF_1. Figure S9, Recall vs. Precision: IG_MHG. Figure S10, Recall vs. Precision: INSULIN. Figure S11, Recall vs. Precision: LACTALBUMIN_LYSOZYME. Figure S12, Recall vs. Precision: LECTIN_LEGUME_BETA. Figure S13, Recall vs. Precision: PA2_HIS. Figure S14, Recall vs. Precision: PROTEIN_KINASE_ST. Figure S15, Recall vs. Precision: PROTEIN_KINASE_TYR. Figure S16, Recall vs. Precision: RNASE_PANCREATIC. Figure S17, Recall vs. Precision: SOD_CU_ZN_1. Figure S18, Recall vs. Precision: TRYPSIN_HIS. Figure S19, Recall vs. Precision: TRYPSIN _SER. Figure S20, Recall vs. Precision: ZINC_PROTEASE. (ZIP) [file pone.0091240.s001.zip › Figure_S1_ADH_SHORT.tiff]

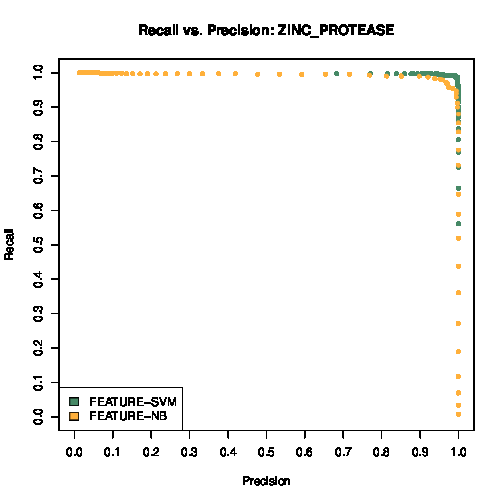

Supplement: File S1 — This file contains Figures S1–S20, which are recall vs. precision graphs for the 20 models analyzed in the paper. Figure S1, Recall vs. Precision: ADH_SHORT. Figure S2, Recall vs. Precision: Alpha_CA_1. Figure S3, Recall vs. Precision: ASP_PROTEASE. Figure S4, Recall vs. Precision: ATPASE_ALPHA_BETA. Figure S5, Recall vs. Precision: CARBOXYLESTERASE_B_2. Figure S6, Recall vs. Precision: CYTOCHROME_P450. Figure S7, Recall vs. Precision: EF_HAND. Figure S8, Recall vs. Precision: EGF_1. Figure S9, Recall vs. Precision: IG_MHG. Figure S10, Recall vs. Precision: INSULIN. Figure S11, Recall vs. Precision: LACTALBUMIN_LYSOZYME. Figure S12, Recall vs. Precision: LECTIN_LEGUME_BETA. Figure S13, Recall vs. Precision: PA2_HIS. Figure S14, Recall vs. Precision: PROTEIN_KINASE_ST. Figure S15, Recall vs. Precision: PROTEIN_KINASE_TYR. Figure S16, Recall vs. Precision: RNASE_PANCREATIC. Figure S17, Recall vs. Precision: SOD_CU_ZN_1. Figure S18, Recall vs. Precision: TRYPSIN_HIS. Figure S19, Recall vs. Precision: TRYPSIN _SER. Figure S20, Recall vs. Precision: ZINC_PROTEASE. (ZIP) [file pone.0091240.s001.zip › Figure_S20_ZINC_PROTEASE.tiff]

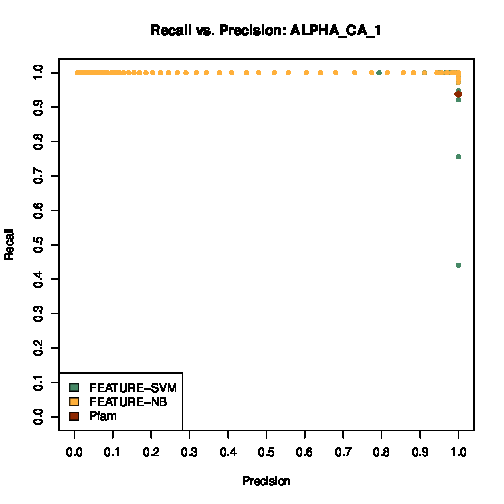

Supplement: File S1 — This file contains Figures S1–S20, which are recall vs. precision graphs for the 20 models analyzed in the paper. Figure S1, Recall vs. Precision: ADH_SHORT. Figure S2, Recall vs. Precision: Alpha_CA_1. Figure S3, Recall vs. Precision: ASP_PROTEASE. Figure S4, Recall vs. Precision: ATPASE_ALPHA_BETA. Figure S5, Recall vs. Precision: CARBOXYLESTERASE_B_2. Figure S6, Recall vs. Precision: CYTOCHROME_P450. Figure S7, Recall vs. Precision: EF_HAND. Figure S8, Recall vs. Precision: EGF_1. Figure S9, Recall vs. Precision: IG_MHG. Figure S10, Recall vs. Precision: INSULIN. Figure S11, Recall vs. Precision: LACTALBUMIN_LYSOZYME. Figure S12, Recall vs. Precision: LECTIN_LEGUME_BETA. Figure S13, Recall vs. Precision: PA2_HIS. Figure S14, Recall vs. Precision: PROTEIN_KINASE_ST. Figure S15, Recall vs. Precision: PROTEIN_KINASE_TYR. Figure S16, Recall vs. Precision: RNASE_PANCREATIC. Figure S17, Recall vs. Precision: SOD_CU_ZN_1. Figure S18, Recall vs. Precision: TRYPSIN_HIS. Figure S19, Recall vs. Precision: TRYPSIN _SER. Figure S20, Recall vs. Precision: ZINC_PROTEASE. (ZIP) [file pone.0091240.s001.zip › Figure_S2_ALPHA_CA_1.tiff]

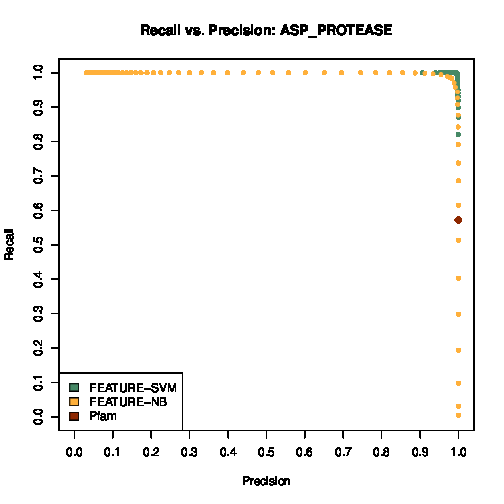

Supplement: File S1 — This file contains Figures S1–S20, which are recall vs. precision graphs for the 20 models analyzed in the paper. Figure S1, Recall vs. Precision: ADH_SHORT. Figure S2, Recall vs. Precision: Alpha_CA_1. Figure S3, Recall vs. Precision: ASP_PROTEASE. Figure S4, Recall vs. Precision: ATPASE_ALPHA_BETA. Figure S5, Recall vs. Precision: CARBOXYLESTERASE_B_2. Figure S6, Recall vs. Precision: CYTOCHROME_P450. Figure S7, Recall vs. Precision: EF_HAND. Figure S8, Recall vs. Precision: EGF_1. Figure S9, Recall vs. Precision: IG_MHG. Figure S10, Recall vs. Precision: INSULIN. Figure S11, Recall vs. Precision: LACTALBUMIN_LYSOZYME. Figure S12, Recall vs. Precision: LECTIN_LEGUME_BETA. Figure S13, Recall vs. Precision: PA2_HIS. Figure S14, Recall vs. Precision: PROTEIN_KINASE_ST. Figure S15, Recall vs. Precision: PROTEIN_KINASE_TYR. Figure S16, Recall vs. Precision: RNASE_PANCREATIC. Figure S17, Recall vs. Precision: SOD_CU_ZN_1. Figure S18, Recall vs. Precision: TRYPSIN_HIS. Figure S19, Recall vs. Precision: TRYPSIN _SER. Figure S20, Recall vs. Precision: ZINC_PROTEASE. (ZIP) [file pone.0091240.s001.zip › Figure_S3_ASP_PROTEASE.tiff]

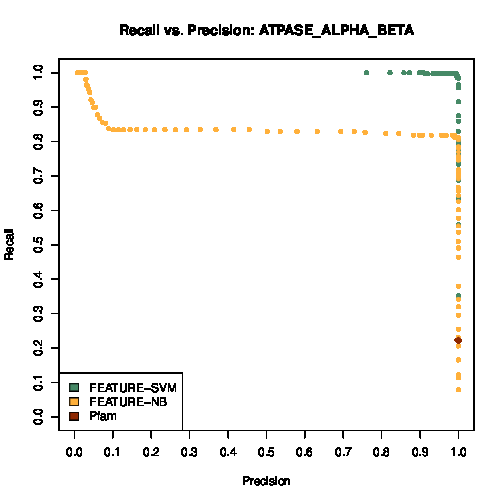

Supplement: File S1 — This file contains Figures S1–S20, which are recall vs. precision graphs for the 20 models analyzed in the paper. Figure S1, Recall vs. Precision: ADH_SHORT. Figure S2, Recall vs. Precision: Alpha_CA_1. Figure S3, Recall vs. Precision: ASP_PROTEASE. Figure S4, Recall vs. Precision: ATPASE_ALPHA_BETA. Figure S5, Recall vs. Precision: CARBOXYLESTERASE_B_2. Figure S6, Recall vs. Precision: CYTOCHROME_P450. Figure S7, Recall vs. Precision: EF_HAND. Figure S8, Recall vs. Precision: EGF_1. Figure S9, Recall vs. Precision: IG_MHG. Figure S10, Recall vs. Precision: INSULIN. Figure S11, Recall vs. Precision: LACTALBUMIN_LYSOZYME. Figure S12, Recall vs. Precision: LECTIN_LEGUME_BETA. Figure S13, Recall vs. Precision: PA2_HIS. Figure S14, Recall vs. Precision: PROTEIN_KINASE_ST. Figure S15, Recall vs. Precision: PROTEIN_KINASE_TYR. Figure S16, Recall vs. Precision: RNASE_PANCREATIC. Figure S17, Recall vs. Precision: SOD_CU_ZN_1. Figure S18, Recall vs. Precision: TRYPSIN_HIS. Figure S19, Recall vs. Precision: TRYPSIN _SER. Figure S20, Recall vs. Precision: ZINC_PROTEASE. (ZIP) [file pone.0091240.s001.zip › Figure_S4_ATPASE_ALPHA_BETA.tiff]

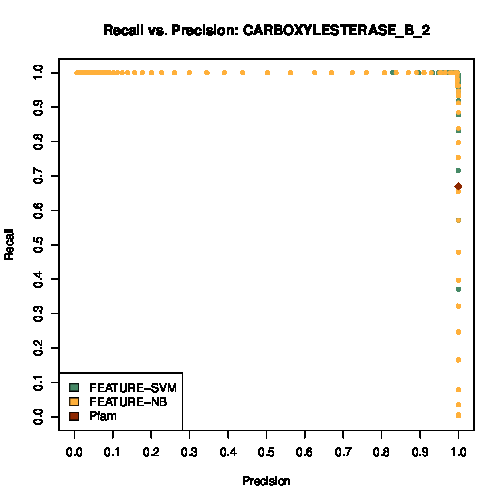

Supplement: File S1 — This file contains Figures S1–S20, which are recall vs. precision graphs for the 20 models analyzed in the paper. Figure S1, Recall vs. Precision: ADH_SHORT. Figure S2, Recall vs. Precision: Alpha_CA_1. Figure S3, Recall vs. Precision: ASP_PROTEASE. Figure S4, Recall vs. Precision: ATPASE_ALPHA_BETA. Figure S5, Recall vs. Precision: CARBOXYLESTERASE_B_2. Figure S6, Recall vs. Precision: CYTOCHROME_P450. Figure S7, Recall vs. Precision: EF_HAND. Figure S8, Recall vs. Precision: EGF_1. Figure S9, Recall vs. Precision: IG_MHG. Figure S10, Recall vs. Precision: INSULIN. Figure S11, Recall vs. Precision: LACTALBUMIN_LYSOZYME. Figure S12, Recall vs. Precision: LECTIN_LEGUME_BETA. Figure S13, Recall vs. Precision: PA2_HIS. Figure S14, Recall vs. Precision: PROTEIN_KINASE_ST. Figure S15, Recall vs. Precision: PROTEIN_KINASE_TYR. Figure S16, Recall vs. Precision: RNASE_PANCREATIC. Figure S17, Recall vs. Precision: SOD_CU_ZN_1. Figure S18, Recall vs. Precision: TRYPSIN_HIS. Figure S19, Recall vs. Precision: TRYPSIN _SER. Figure S20, Recall vs. Precision: ZINC_PROTEASE. (ZIP) [file pone.0091240.s001.zip › Figure_S5_CARBOXYLESTERASE_B_2.tiff]

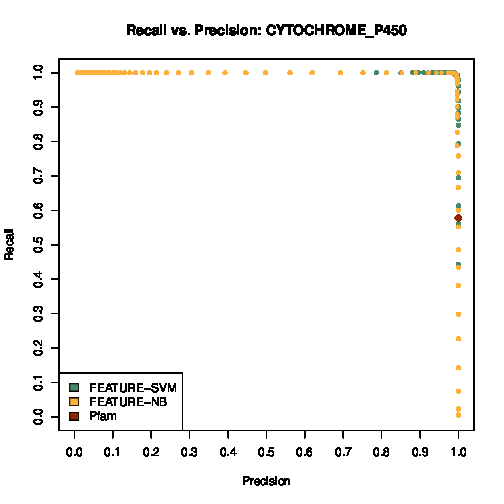

Supplement: File S1 — This file contains Figures S1–S20, which are recall vs. precision graphs for the 20 models analyzed in the paper. Figure S1, Recall vs. Precision: ADH_SHORT. Figure S2, Recall vs. Precision: Alpha_CA_1. Figure S3, Recall vs. Precision: ASP_PROTEASE. Figure S4, Recall vs. Precision: ATPASE_ALPHA_BETA. Figure S5, Recall vs. Precision: CARBOXYLESTERASE_B_2. Figure S6, Recall vs. Precision: CYTOCHROME_P450. Figure S7, Recall vs. Precision: EF_HAND. Figure S8, Recall vs. Precision: EGF_1. Figure S9, Recall vs. Precision: IG_MHG. Figure S10, Recall vs. Precision: INSULIN. Figure S11, Recall vs. Precision: LACTALBUMIN_LYSOZYME. Figure S12, Recall vs. Precision: LECTIN_LEGUME_BETA. Figure S13, Recall vs. Precision: PA2_HIS. Figure S14, Recall vs. Precision: PROTEIN_KINASE_ST. Figure S15, Recall vs. Precision: PROTEIN_KINASE_TYR. Figure S16, Recall vs. Precision: RNASE_PANCREATIC. Figure S17, Recall vs. Precision: SOD_CU_ZN_1. Figure S18, Recall vs. Precision: TRYPSIN_HIS. Figure S19, Recall vs. Precision: TRYPSIN _SER. Figure S20, Recall vs. Precision: ZINC_PROTEASE. (ZIP) [file pone.0091240.s001.zip › Figure_S6_CYTOCHROME_P450.tiff]

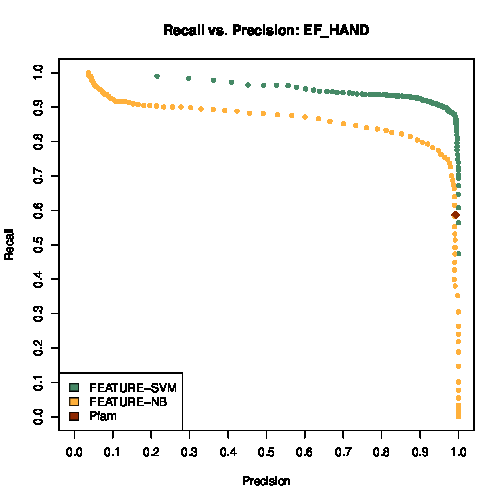

Supplement: File S1 — This file contains Figures S1–S20, which are recall vs. precision graphs for the 20 models analyzed in the paper. Figure S1, Recall vs. Precision: ADH_SHORT. Figure S2, Recall vs. Precision: Alpha_CA_1. Figure S3, Recall vs. Precision: ASP_PROTEASE. Figure S4, Recall vs. Precision: ATPASE_ALPHA_BETA. Figure S5, Recall vs. Precision: CARBOXYLESTERASE_B_2. Figure S6, Recall vs. Precision: CYTOCHROME_P450. Figure S7, Recall vs. Precision: EF_HAND. Figure S8, Recall vs. Precision: EGF_1. Figure S9, Recall vs. Precision: IG_MHG. Figure S10, Recall vs. Precision: INSULIN. Figure S11, Recall vs. Precision: LACTALBUMIN_LYSOZYME. Figure S12, Recall vs. Precision: LECTIN_LEGUME_BETA. Figure S13, Recall vs. Precision: PA2_HIS. Figure S14, Recall vs. Precision: PROTEIN_KINASE_ST. Figure S15, Recall vs. Precision: PROTEIN_KINASE_TYR. Figure S16, Recall vs. Precision: RNASE_PANCREATIC. Figure S17, Recall vs. Precision: SOD_CU_ZN_1. Figure S18, Recall vs. Precision: TRYPSIN_HIS. Figure S19, Recall vs. Precision: TRYPSIN _SER. Figure S20, Recall vs. Precision: ZINC_PROTEASE. (ZIP) [file pone.0091240.s001.zip › Figure_S7_EF_HAND.tiff]

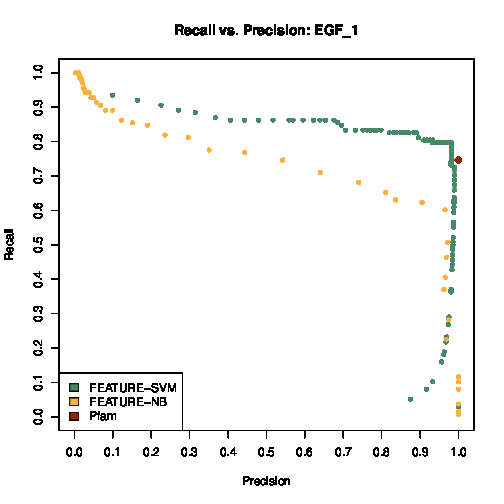

Supplement: File S1 — This file contains Figures S1–S20, which are recall vs. precision graphs for the 20 models analyzed in the paper. Figure S1, Recall vs. Precision: ADH_SHORT. Figure S2, Recall vs. Precision: Alpha_CA_1. Figure S3, Recall vs. Precision: ASP_PROTEASE. Figure S4, Recall vs. Precision: ATPASE_ALPHA_BETA. Figure S5, Recall vs. Precision: CARBOXYLESTERASE_B_2. Figure S6, Recall vs. Precision: CYTOCHROME_P450. Figure S7, Recall vs. Precision: EF_HAND. Figure S8, Recall vs. Precision: EGF_1. Figure S9, Recall vs. Precision: IG_MHG. Figure S10, Recall vs. Precision: INSULIN. Figure S11, Recall vs. Precision: LACTALBUMIN_LYSOZYME. Figure S12, Recall vs. Precision: LECTIN_LEGUME_BETA. Figure S13, Recall vs. Precision: PA2_HIS. Figure S14, Recall vs. Precision: PROTEIN_KINASE_ST. Figure S15, Recall vs. Precision: PROTEIN_KINASE_TYR. Figure S16, Recall vs. Precision: RNASE_PANCREATIC. Figure S17, Recall vs. Precision: SOD_CU_ZN_1. Figure S18, Recall vs. Precision: TRYPSIN_HIS. Figure S19, Recall vs. Precision: TRYPSIN _SER. Figure S20, Recall vs. Precision: ZINC_PROTEASE. (ZIP) [file pone.0091240.s001.zip › Figure_S8_EGF_1.tiff]

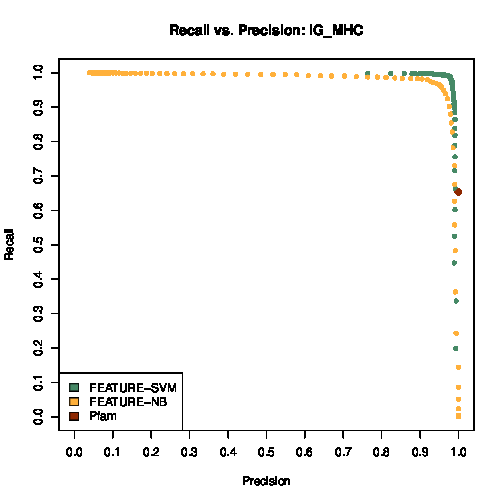

Supplement: File S1 — This file contains Figures S1–S20, which are recall vs. precision graphs for the 20 models analyzed in the paper. Figure S1, Recall vs. Precision: ADH_SHORT. Figure S2, Recall vs. Precision: Alpha_CA_1. Figure S3, Recall vs. Precision: ASP_PROTEASE. Figure S4, Recall vs. Precision: ATPASE_ALPHA_BETA. Figure S5, Recall vs. Precision: CARBOXYLESTERASE_B_2. Figure S6, Recall vs. Precision: CYTOCHROME_P450. Figure S7, Recall vs. Precision: EF_HAND. Figure S8, Recall vs. Precision: EGF_1. Figure S9, Recall vs. Precision: IG_MHG. Figure S10, Recall vs. Precision: INSULIN. Figure S11, Recall vs. Precision: LACTALBUMIN_LYSOZYME. Figure S12, Recall vs. Precision: LECTIN_LEGUME_BETA. Figure S13, Recall vs. Precision: PA2_HIS. Figure S14, Recall vs. Precision: PROTEIN_KINASE_ST. Figure S15, Recall vs. Precision: PROTEIN_KINASE_TYR. Figure S16, Recall vs. Precision: RNASE_PANCREATIC. Figure S17, Recall vs. Precision: SOD_CU_ZN_1. Figure S18, Recall vs. Precision: TRYPSIN_HIS. Figure S19, Recall vs. Precision: TRYPSIN _SER. Figure S20, Recall vs. Precision: ZINC_PROTEASE. (ZIP) [file pone.0091240.s001.zip › Figure_S9_IG_MHC.tiff]
